# Multivariate Deep Learning Classification of Alzheimer’s Disease Based on Hierarchical Partner Matching Independent Component Analysis

**DOI:** 10.3389/fnagi.2018.00417

**Published:** 2018-12-17

**Authors:** Jianping Qiao, Yingru Lv, Chongfeng Cao, Zhishun Wang, Anning Li

**Affiliations:** ^1^Shandong Province Key Laboratory of Medical Physics and Image Processing Technology, Institute of Data Science and Technology, School of Physics and Electronics, Shandong Normal University, Jinan, China; ^2^Department of Radiology, Huashan Hospital, Fudan University, Shanghai, China; ^3^Department of Emergency, Jinan Central Hospital Affiliated to Shandong University, Jinan, China; ^4^Department of Psychiatry, Columbia University, New York, NY, United States; ^5^Department of Radiology, Qilu Hospital of Shandong University, Jinan, China

**Keywords:** Alzheimer’s disease, independent component analysis, granger causality, brain network, deep learning

## Abstract

Machine learning and pattern recognition have been widely investigated in order to look for the biomarkers of Alzheimer’s disease (AD). However, most existing methods extract features by seed-based correlation, which not only requires prior information but also ignores the relationship between resting state functional magnetic resonance imaging (rs-fMRI) voxels. In this study, we proposed a deep learning classification framework with multivariate data-driven based feature extraction for automatic diagnosis of AD. Specifically, a three-level hierarchical partner matching independent components analysis (3LHPM-ICA) approach was proposed first in order to address the issues in spatial individual ICA, including the uncertainty of the numbers of components, the randomness of initial values, and the correspondence of ICs of multiple subjects, resulting in stable and reliable ICs which were applied as the intrinsic brain functional connectivity (FC) features. Second, Granger causality (GC) was utilized to infer directional interaction between the ICs that were identified by the 3LHPM-ICA method and extract the effective connectivity features. Finally, a deep learning classification framework was developed to distinguish AD from controls by fusing the functional and effective connectivities. A resting state fMRI dataset containing 34 AD patients and 34 normal controls (NCs) was applied to the multivariate deep learning platform, leading to a classification accuracy of 95.59%, with a sensitivity of 97.06% and a specificity of 94.12% with leave-one-out cross validation (LOOCV). The experimental results demonstrated that the measures of neural connectivities of ICA and GC followed by deep learning classification represented the most powerful methods of distinguishing AD clinical data from NCs, and these aberrant brain connectivities might serve as robust brain biomarkers for AD. This approach also allows for expansion of the methodology to classify other psychiatric disorders.

## Introduction

Alzheimer’s disease (AD) is a chronic neurodegenerative disease characterized by cognitive and intellectual deficits that are serious enough to interfere with daily life. It usually starts slowly and worsens over time by destroying brain cells, leading to memory loss, problems performing familiar tasks, vision problems, thinking, reasoning, and personality changes (Burns and Iliffe, 2009; Querfurth and LaFerla, 2010). Gradually, bodily functions are lost, ultimately leading to death (Alzheimer’s Association, 2011). With the aging of the world population, AD has become a serious problem to the health the elderly people and a huge burden to the healthcare system. Nowadays, AD can only be slowed down and delayed by drugs, and effective treatment remains elusive (Jack et al., 2008). The diagnosis of AD is usually based on cognitive impairments relating to daily activities or positive physiopathologic markers of AD, such as an abnormal level of amyloid beta and/or tau in the cerebrospinal fluid (Dubois et al., 2014). Therefore, it is of great interest to develop objective biomarkers of AD patients with the help of neuroimaging studies in order to assist AD clinical diagnosis and monitor the efficacy of treatment.

Brain imaging technology, combined with advanced signal processing approaches, has been actively applied to investigate the underlying biological or neurological mechanisms and to discover differences between AD patients and normal controls (NCs) for AD diagnosis or prognosis (Mirzaei et al., 2016). Positron emission tomography (PET) accessed the pathophysiologic markers of AD as reductions of glucose metabolism in the parietal, posterior cingulate and temporal brain regions of AD patients (Diehl et al., 2004). Additionally, high resolution structural magnetic resonance imaging (sMRI) studies have shown that neuroimaging measurements included cortical thickness (Thompson et al., 2004; Lerch et al., 2008; Desikan et al., 2009; Dickerson et al., 2009), gray matter density (Dai et al., 2012; Liu M. et al., 2015; Liu et al., 2016), hippocampal volume and shape (Colliot et al., 2008; Fan et al., 2008; Hua et al., 2008; Chupin et al., 2009; Tsao et al., 2017). Histogram characteristics of regions of interest (ROIs) in the whole brain (Magnin et al., 2009) could be investigated as brain features for the classification between AD and NC. Furthermore, the measures of diffusion tensor imaging (DTI) such as fractional anisotropy (FA) and mean diffusivity (MD), which indicated white matter (WM) fiber tract integrity, have been reported to discriminate AD from NC (Dyrba et al., 2013). Another study reported that the WM tracts connecting brain regions defined by 41 Brodmann areas were reconstructed as the brain connectivity network and the graphs of the connectivity matrices were described as feature vectors for the classification of AD (Ebadi et al., 2017). Moreover, the absolute and relative spectral power, distribution of spectral power, and measures of spatial synchronization were calculated from recordings of the electroencephalography (EEG) by following classification models for the clinical diagnosis of AD (Lehmann et al., 2007). The lagged linear connectivity of predefined ROIs was also used as an EEG marker of AD (Babiloni et al., 2016; Triggiani et al., 2017).

Besides, resting state functional MRI (rs-fMRI) combined with machine learning has played an important role in identifying biomarkers of AD. Various classification features of AD have been detected in previous studies, such as the amplitude of low frequency fluctuations (Dai et al., 2012) or hippocampal correlation of low frequency components (Li et al., 2002), regional homogeneity (Dai et al., 2012), functional correlation strength of 90 ROIs in terms of the automated anatomical labeling (AAL) atlas (Dai et al., 2012), whole-brain (Chen et al., 2011; Ju et al., 2017) or selected regional (Wang K. et al., 2006) functional correlation connectivity matrices based on AAL or other atlas (Khazaee et al., 2016), covariance connectivity matrices (Challis et al., 2015), and graph-theoretical measures (Dyrba et al., 2015; Khazaee et al., 2015, 2017). However, most of the existing studies focus on seed-based correlation analysis which needed a prior (such as atlas) and ignored the relationship between voxels of brain images. The performance of the seed-based correlation methods may be unstable due to the different seeds or atlas as well as the error of the registration processing (Wang et al., 2009; Zalesky et al., 2010; Craddock et al., 2012). Therefore, as a multivariate data-driven based method, independent component analysis (ICA) was investigated to extract features for automatic classification of AD in the study, which could identify the underlying data structure by counting for the relationship between voxels and without need of prior information.

ICA has been widely applied for analyzing neuroimaging data (Calhoun et al., 2009) and acknowledged as one of the two most commonly used methods in functional connectivity (FC) studies (Zhang and Raichle, 2010). At present, there are two kinds of ICA methods applied to fMRI: individual ICA and group ICA. Previous studies have demonstrated that the AD patients displayed lower FC within the default mode network (DMN) identified by spatial individual ICA (Toussaint et al., 2014) or group ICA (Binnewijzend et al., 2012). A recent study reported that the FC matrices obtained by group ICA and the graph properties can be applied for the classification of AD (de Vos et al., 2018). However, compared with group ICA, the specificity of the individuals can be preserved better in the individual ICA method because a single temporally concatenated data set of all subjects is decomposed into ICs in group ICA. This leads to the possibility that the obtained ICs may not be maximally spatially independent for single subjects and degrades the precision of the identified functional brain network. Therefore, this study focuses on the individual ICA in order to extract the distinguishable features and predict the individuals with AD. However, there are still some problems in individual ICA method. First, the output order of ICs is uncertain, leading to the difficult establishment of the correspondence between the ICs or functional networks of multiple subjects. Second, the number of components must be defined before ICA is performed. Various brain functional networks might be obtained when the specified number is different. Lastly, the FC patterns resulting from multiple implementations of the same ICA algorithm on the same fMRI data may be inconsistent because of the randomness of the initial value in the ICA algorithm.

To address the issues mentioned above, we proposed a three-level hierarchical partner matching ICA (3LHPM-ICA) approach, which could identify the stable and reproducible ICs across multiple individuals. Then the extracted FC features were fused with the effective connectivity matrices computed by Granger causality (GC). Finally, the two-dimensional feature matrices were entered into the deep learning classifier to distinguish AD from NC. The aim of the current study was to detect the underlying fMRI data structure and biomarkers of AD with the multivariate data-driven based feature extraction and deep learning platform by counting for the relationship between voxels without needing prior information.

## Materials and Methods

### Participants

Thirty-four participants with mild AD (17 females, 17 males, mean age 68.64 ± 9.85 years, education 11.47 ± 3.49 years) were recruited from a memory outpatient clinic at the Huashan Hospital of Fudan University. Thirty-four age-matched NCs (13 females, 21 males, mean age 65.55 ± 8.98 years, education 11.31 ± 3.75 years) were recruited by public advertisement to take part in the study. All AD participants fulfilled the following clinical criteria: the National Institute of Neurological and Communicative Disorders and Stroke/Alzheimer’s Disease and Related Disorders Association (NINCDS-ADRDA; McKhann et al., 1984) criteria for AD, Mini Mental State Examination (MMSE) scores between 19 and 23 (inclusive), Clinical Dementia Rating (CDR) scores (Morris, 1993) of 1.0, Hachinski Ischemic Scale (HIS) scores less than 4.0 for the exclusion of vascular dementia and mixed dementia, and there were not any structural abnormalities other than atrophy in MRI scans. A standard diagnostic examination that included physical and neurological examination, medical history taking, extensive neuropsychological assessments and screening laboratory tests, was implemented for all patients. The mean MMSE score of AD group in this study was 21.50 ± 1.61. All NC subjects had normal neurological examinations, with a CDR score of 0 and independently functioning community membership with no history of neurological or psychiatric disorders, cognitive complaints, brain damage or psychoactive medication. All participants were right-handed with ten or more years of education. This study was carried out in accordance with the recommendations of NINCDS-ADRDA, the Institutional Review Board of Huashan Hospital of Fudan University with written informed consent from all subjects. All subjects gave written informed consent in accordance with the Declaration of Helsinki. The protocol was approved by the Institutional Review Board of Huashan Hospital of Fudan University.

### Image Acquisition

Imaging was performed on a Siemens Verio 3.0 Tesla MRI scanner (Siemens, Erlangen, Germany). The head of each participant was snugly fixed by using foam pads to reduce head movements and scanner noise. Participants were instructed to rest with their eyes closed but not to fall asleep during scanning. Resting state fMRI data were acquired using a T2*-weighted echoplanar imaging (EPI) with blood oxygen level dependent (BOLD) contrast pulse sequence. Thirty-three contiguous axial slices were acquired along the anterior commissure-posterior commissure (AC-PC) plane. The acquisition parameters were as follows: matrix = 64 × 64, field of view (FOV) = 20 cm, repetition time (TR) = 2,000 ms, echo time (TE) = 35 ms, voxel size = 3.0 × 3.0 × 4.0 mm^3^, flip angle = 90°, slice thickness = 4 mm. The sequence took 6 min and 40 s, resulting in a total of 200 volumes.

### Image Analysis

#### Preprocessing

All preprocessing steps of the resting state fMRI images were performed with SPM12 (Welcome Department of Imaging Neuroscience, London, United Kingdom) implemented in MATLAB. The functional scans were slice time corrected for the interleaved acquisition, spatially realigned to the first scan to correct for head movements, normalized to the Montreal Neurological Institute (MNI) coordinate system and spatially smoothed with an isotropic 8 mm full-width at half-maximum (FWHM) Gaussian kernel.

#### Functional Connectivity Analysis Based on 3LHPM-ICA

In this study, a 3LHPM-ICA approach was proposed in order to solve the problems of individual ICA method. These included the uncertainty of the output ICs order, the selection of the number of components, and the randomness of the initial value in the ICA algorithm, which could identify the reliable and stable ICs and obtain the intrinsic brain functional networks. Spatial ICA was performed on the preprocessed fMRI images for each participant. The obtained ICs were maps that were maximally spatially independent for each subject and represented the brain functional subnetworks. The mixing matrix represented time courses of the ICs, which represented the changes of the brain functional networks over time.

**Figure 1:**
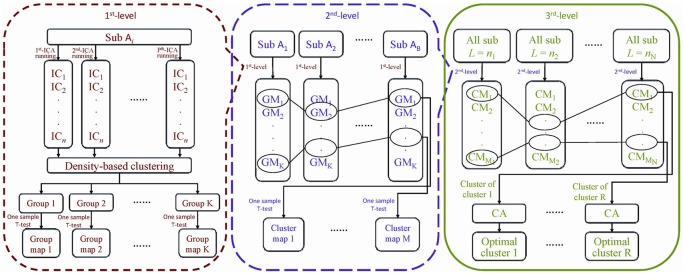
The flowchart of the three-level hierarchical partner matching independent component analysis (3LHPM-ICA) algorithm.

The number of ICs needs to be specified before ICA is performed. One cannot, however, know* a priori* the single number of components to generate with ICA that is “optimal” for the identification of reproducible components across individuals. Therefore, the principles of information criteria were applied to determine the number of sets of ICs in this study. We combined minimum description length (Calhoun et al., 2001) and Akaike’s information criterion (Wang et al., 2011a) to estimate the interval (lower and upper bounds) and step size of the numbers of ICs. Additionally, the initial values of the ICA algorithm are random, meaning that the objective function in the ICA algorithm may fall into a different local extremum. As a result, the inconsistent ICs may be produced when the same ICA algorithm is performed on the same subject with the same number of components. Accordingly, in this study, the spatial ICA algorithm was run several times with the estimated numbers of ICs on each individual subject. Then the correspondence of ICs between different subjects with a set of numbers of ICs was established by the hierarchical partner matching method, which we proposed and published previously (Wang et al., 2011a; Qiao et al., 2015, 2017). In detail, the proposed 3LHPM-ICA approach consists of three levels as follows and its framework is shown in Figure 1.

In the first level, in order to address the problem of the randomness of the initial values in the ICA algorithm, we inputted the fMRI data of each subject and performed spatial ICA by *P* multiplied with the single number of ICs. Then the ICs of the subject (denoted as subject *A*_j_) were clustered by the density-based clustering algorithm which had high efficiency and low complexity (Rodriguez and Laio, 2014). Specifically, each IC was considered as one point in the high dimensional space. The local density of the point and its distance from points of higher density were computed for each data point. Here, the Pearson correlation coefficient was applied to measure the distance between two points. Then, the local density and distance of all points were sorted in descending order. The first *K* points were identified as center points. After that, the distances from all other points to the center points were calculated for group assignment. Finally, a group map (GM) was generated by running one-sample *t*-tests on each group of ICs.

In the second level, in order to solve the problem of the correspondence of ICs across different individuals, the GMs of all the subjects {*A*_1_, *A*_2_, …, *A*_B_} that generated with the same single number of ICs were matched by the partner matching method, which we proposed and published previously (Wang and Peterson, 2008). The Tanimoto distance was used to measure the similarity between GMs. Given a GM_i_ of subject *A*_1_, the indices of spatial similarity between GM_i_ and all the GMs of subject *A*_2_ were calculated. The GM_j_ of subject *A*_2_ was selected, which had the maximum similarity index with GM_i_ of subject *A*_1_ among all the GMs of subject *A*_2_. After that, the similarity indices between GM_j_ of subject *A*_2_ and all the GMs of subject *A*_1_ were calculated. The GM_k_ of subject *A*_1_ was selected which had the maximum similarity index with GM_j_ of subject *A*_2_ among all the GMs of subject *A*_1_. If *k* = *i*, then the matching was bidirectional, and we considered GM_i_ of subject *A*_1_ and GM_j_ of subject *A*_2_ to be partner matched. This procedure was repeated to find all pairs of GMs that are bidirectionally matched between subject *A*_1_ and *A*_2_. Similarly, the partner matching method was performed to identify matching GMs across all the subjects. A collection of GMs that match across subjects was termed as a *cluster*. Finally, a cluster map (CM) was generated by running one-sample *t*-tests on each cluster of GMs, which represented a spatial pattern that tends to be present across subjects.

In the third level, in order to figure out the correspondence of ICs across different numbers, the CMs of all the subjects that generated with the estimated multiple numbers of ICs *L* = {*n*_1_, *n*_2_, …, *n*_N_} were clustered by the partner matching method, identifying corresponding CMs across the different sets that were obtained with different numbers of ICs. For each cluster of CMs, the cluster with the highest Cronbach’s Alpha was selected as the optimal cluster. The CMs were derived from GMs and GMs were derived from ICs, thus the most reliable and stable ICs could be obtained by backward tracing from optimal clusters.

#### Effective Connectivity Analysis Based on Granger Causality

GC has been widely applied to assess brain effective connectivity in fMRI data analysis. Compared with the structural equation model and dynamic causal model, GC analysis is very consistent with the actual situation because it considers time and does not require any prior knowledge (Goebel et al., 2003; Cohen Kadosh et al., 2016). In this study, we computed the GC index (GCI) to assess the causal influence between the ICs that were identified by the 3LHPM-ICA method.

Let *X*(*t*) denote the zero-mean vector time course of an ICs within region *X*, and *Y(t)* denote the zero-mean vector time course of another IC within region *Y*. Then *X*(*t*) can be estimated by applying an autoregressive (AR) model of order *P* as follows:

(1) $$X(t)=\sum_{\text{i }=\;1}^{P}\alpha_{\text{i}}X(t-\text{i})+\varepsilon_{X}$$

where *α*_i_ are coefficients of the *AR* model and *ε*_X_ is the zero-mean residual. The *Y*(*t*) is then added into the above AR model and *X*(*t*) can be estimated by

(2) $$X(t)=\sum_{\text{i }=\;1}^{P}\alpha_{\text{i}}X(t-\text{i})+\sum_{\text{j }=\;1}^{P}\beta_{\text{j}}Y(t-\text{j})\varepsilon_{XY}$$

where *β*_j_ are coefficients of the *AR* model and *ε*_XY_ is the new zero-mean residual. To assess whether the addition of *Y*(*t*) improves the prediction compared with the use of *X*(*t*) alone, the GCI from *Y* to *X* can be calculated by

(3) $$GCI_{Y\to X}=1-\frac{var(\varepsilon_{XY})}{var(\varepsilon_{X})}$$

where *var*(*ε*_XY_) and (*ε*_X_) are the variance of the estimation errors or residuals *ε*_XY_ and *ε*_X_, respectively. If *GCI*_(Y→X)_ is greater than zero, the addition of the previous values of *Y*(*t*) into the right side of Equation (1) significantly improves the prediction of the current values of *X*(*t*) and we can deem that *Y*(*t*) Granger caused *X*(*t*), that is, region *Y* has a causal influence and directional interaction to region *X*.

In this way, a GCI matrix was obtained by repeating the above procedure to all ICs for each subject. In the GCI effective connectivity matrix, rows and columns of the matrix represented different ICs. Each cell of the matrix represented a distinct connection between two ICs corresponding to specific row and column. The diagonal value of the matrix was *NaN* because there was no meaningful directional interaction from one IC to the same one. The GCI matrices of all subjects were computed, which would be applied as an effective feature in the following classifier.

#### Feature Fusion and Classification

**Figure 2:**
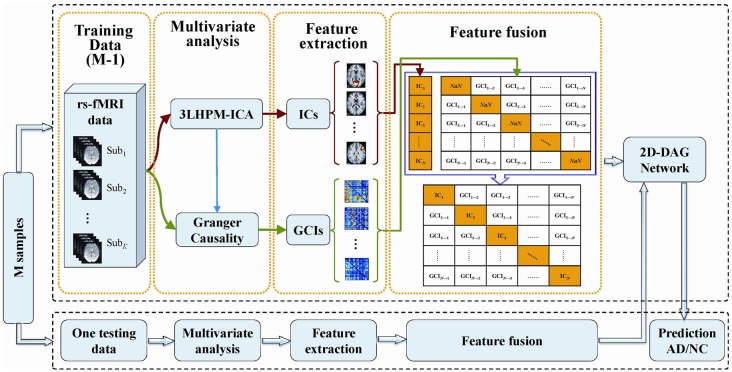
The framework of the proposed deep learning classification algorithm based on 3LHPM-ICA and Granger causality (GC).

The deep learning classification framework in this study consists of four steps: multivariate analysis, feature extraction, feature fusion and directed acyclic graph (DAG) network, as shown in Figure 2. The details can be stated as follows. First, reproducible ICs were obtained by performing 3LHPM-ICA on training resting state fMRI data. Then the GCIs were computed to infer directional interaction between these brain regions by extracting the time course of each IC within each pattern. Second, the z-score maps of the reliable ICs were then entered into a two-sample *t*-test model implemented in the SPM12 factorial module to detect group difference of the FC between AD and NC. The ROIs with significant differences (*p* < 0.05, uncorrected) between the two groups of the training set were extracted as FC features for the pattern recognition analyses. In addition, GC matrices computed by the time course of significant ICs were selected as effective connectivity features. Third, functional and effective connectivity features were fused by replacing the diagonal values *NaN* in the GC matrices as IC features. In this way, a matrix feature was obtained for each subject. Finally, the two-dimensional characteristic matrices of the training data were inputted into a deep learning classifier model. Given test fMRI data, the same steps were conducted and a feature matrix was entered into the pretrained network for the prediction of AD/NC. A leave-one-out cross-validation (LOOCV) strategy was applied to evaluate the performance of the classifier.

**Figure 3:**
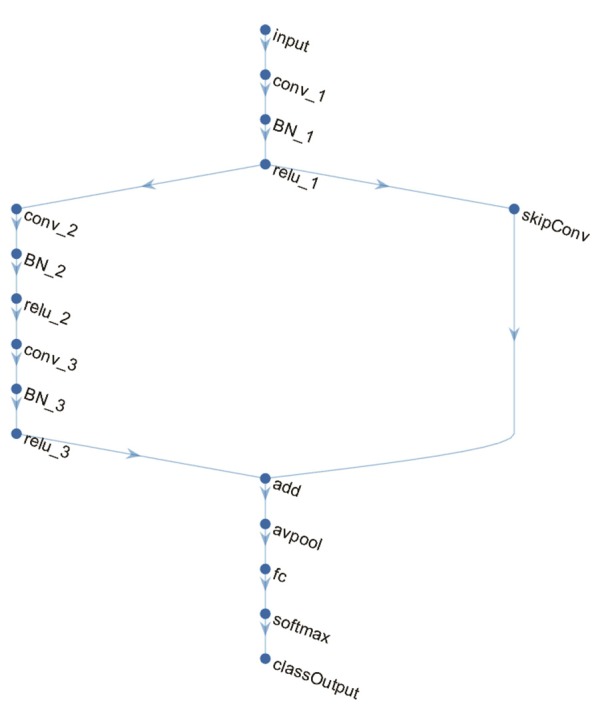
The architecture of the directed acyclic graph (DAG) network.

A DAG network is a deep learning method which has its layers arranged as a DAG and a more complex architecture where layers can have inputs from, or outputs to, multiple layers. In this study, we implemented the DAG network for deep learning with the neural network toolbox in MATLAB R2018a, as shown in Figure 3, which consisted of a main branch with layers connected sequentially and a shortcut connection that enabled the parameter gradients to flow more easily from the output layer to the earlier layers of the network. The main branch contained an image input layer, three convolutional layers, three batch normalization layers, three rectified linear unit (ReLU) layers, an average pooling layer, a fully connected layer, a softmax layer and classification layer. The shortcut connection contained a single one-by-one convolutional layer that had an added benefit of not adding any extra parameters or computational complexity. Batch normalization layers between convolutional layers and ReLU layers normalized the activations and gradients propagating through a network, resulting in speeding up network training and reducing the sensitivity to network initialization. The average pooling layer was applied as a down-sampling operation that reduced the spatial size of the feature map and removed redundant spatial information.

## Results

### ICA-Based Functional Connectivity

**Figure 4:**
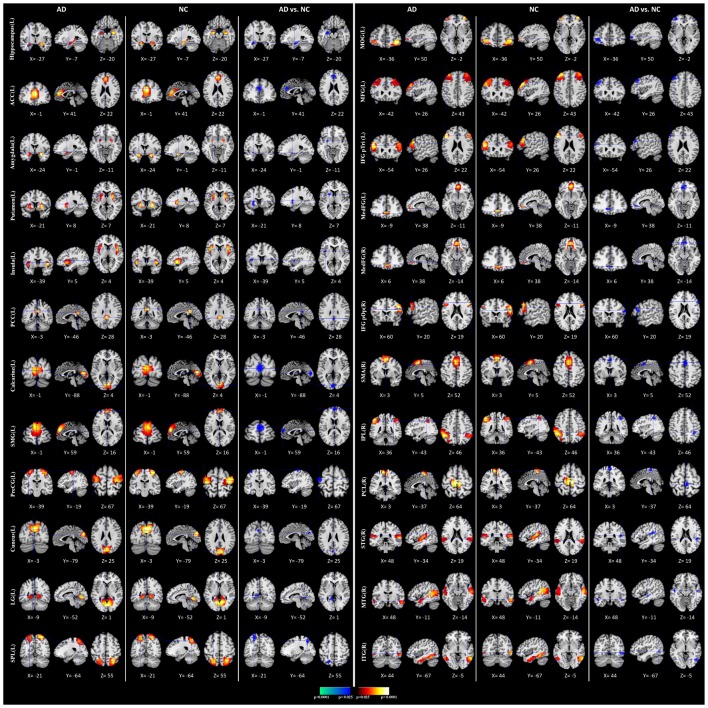
Comparisons of functional connectivity (FC) between Alzheimer’s disease (AD) and normal controls (NCs). The first and fourth columns of three display the random-effect group connectivity maps detected from the AD. Within each column of three, the first column is a coronal view, the second is a sagittal view, and the third is an axial view. The second and fifth columns of three display the group connectivity maps detected from the NCs. Each row displays one group connectivity map generated by applying a one-sample *t*-test to the clusters of ICs. Any two group connectivity maps within the same row across the first three and second three columns (as well as the fourth three and fifth three columns) are significantly similar to one another in their spatial configurations. The third and sixth columns of three display t-contrast maps comparing the group connectivity maps from the AD and control participants. MFG, middle frontal gyrus; MedFG, medial frontal gyrus; SMG, superior medial gyrus; MOG, middle orbital gyrus; IFG pOp, inferior frontal gyrus (p. Opercularis); IFG pTri, inferior frontal gyrus (p. Triangularis); SMA, supplementary motor area; ACC, anterior cingulate cortex; PCC, posterior cingulate cortex; SPL, superior parietal lobule, IPL, inferior parietal lobule; PCL, paracentral lobule; STG, superior temporal gyrus; MTG, middle temporal gyrus; ITG, inferior temporal gyrus; PreCG, precentral gyrus; LG, lingual gyrus.

We performed the 3LHPM-ICA method on the training fMRI data. The numbers of components were set to be 20 to 130, with increments of 10 which were determined by information criteria. In the first level, we performed 10 times ICA with the single number of ICs on the fMRI data of each subject. The first K points were identified as center points in the density-based clustering algorithm. The *K* was set to be *n* plus 10 experimentally, where n is the number of ICs. In the second level, we performed the partner matching method on the training subjects with the same single number of ICs. The numbers of the CMs were 29, 36, 46, 55, 62, 69, 77, 86, 95, 102, 113 and 122, while the numbers of the ICs were 20, 30, 40, 50, 60, 70, 80, 90, 100, 110, 120 and 130, respectively. In the third level, 27 cluster of clusters were obtained after performing the partner matching method. Three artifactual cluster of clusters were excluded. Finally, 24 clusters of ICs that were significantly reproducible in their spatial patterns across individuals were identified. The general linear model in SPM was utilized to perform a one-sample *t*-test on each of the clusters to generate IC maps that represented FC features. After that, the reproducible ICs of AD and NC were compared in a second-level random effects analysis, covarying with age and sex. Compared with NC, FC in AD was significantly decreased in various cortical and subcortical areas related to memory, emotion and cognition, including the middle frontal gyrus (MFG), superior medial gyrus (SMG), middle orbital gyrus (MOG), inferior frontal gyrus (IFG), supplementary motor area (SMA), medial frontal gyrus (MedFG), hippocampus, insula, putamen, anterior cingulate cortex (ACC), posterior cingulate cortex (PCC), superior parietal lobule (SPL), superior temporal gyrus (STG), and middle temporal gyrus (MTG; Figure 4, Table 1).

**Table 1:** Location and comparisons of independent component (IC) maps between Alzheimer’s disease (AD) and normal control (NC).

Brain areas	Location		Peak location			textitT statistic
	Side	BA	*x*	textity	textitz	
AD vs. NC (negative)						
Middle frontal gyrus	L	8	−42	26	43	−4.06
Superior medial gyrus	L	10	−1	59	16	−4.05
Calcarine gyrus	L	17	−1	−88	4	−4.00
Middle orbital gyrus	L	10	−36	50	−2	−3.67
Inferior frontal gyrus (p. Opercularis)	R	44	60	20	19	−3.48
Inferior frontal gyrus (p. Triangularis)	L	45	−54	26	22	−3.28
Supplementary motor area	R	6	3	5	52	−3.03
Precentral gyrus	L	6	−39	−19	67	−3.08
Medial frontal gyrus	L	11	−9	38	−11	−3.97
	R	11	6	38	−14	−3.35
Insula	L	13	−39	5	4	−2.56
Anterior cingulate cortex	L	32	−1	41	22	−6.55
Posterior cingulate cortex	L	23	−3	−46	28	−2.94
Hippocampus	L	54	−27	−7	−20	−4.60
Amygdala	L	53	−24	−1	−11	−3.86
Putamen	L	49	−21	8	7	−2.92
Cuneus	L	18	−3	−79	25	−2.48
Lingual gyrus	L	18	−9	−52	1	−2.84
Superior parietal lobule	L	7	−21	−64	55	−2.63
Inferior parietal lobule	R	7	36	−43	46	−3.41
Paracentral lobule	R	4	3	−37	64	−3.11
Superior temporal gyrus	R	22	48	−34	19	−3.23
Middle temporal gyrus	R	22	48	−11	−14	−3.35
Inferior temporal gyrus	R	20	44	−67	−5	−2.35

*All coordinates are in the Montreal Neurological Institute (MNI) ICBM 152 template*.

### GC Based Effective Connectivity

The effective connectivity was measured by computing the GC of time courses of 24 ICs identified by 3LHPM-ICA. The 24 × 24 GCI matrix was obtained for each subject. The diagonal of the GCI matrix was set to be *NaN* because there is no meaning for the GC from brain area *X* to itself. Finally, the functional and effective connectivity features were fused by replacing the diagonal values of the GCI matrix with IC values in the *z*-score IC maps.

### Classification

We applied the DAG network for deep learning to classify and predict the AD/NC. The image size at the input layer in Figure 3 was 24 × 24 × 1. The filter size in the convolutional layer “*conv_1*” was 5 × 5. The number of filters was 16, which represented the number of neurons that connect to the same region of the input. The filter size of “*conv_2*” and “*conv_3*” were 3 × 3 with 32 filters. The window size in the average pooling layer “*avpool*” was 3 × 3 with stride (or step size) 2 × 2. The filter size in the convolutional layer of the shortcut connection “*skipConv*” was 1 × 1 with 32 filters. The training lasted for 20 epochs. The batch size was 20. The iteration per epoch was three and the total iteration was 60. The initial learning rate was set to be 0.01. The learning rate was multiplied by a factor every time a certain number of epochs had passed. The multiplicative factor was 0.1 and the number of epochs between multiplications was 10. The output was a 1 × 2 vector containing the probabilities of the test data belonging to AD or NC.

In every fold of LOOCV, the number of the training data was 67 and the last one was used as testing data. In the training stage, we performed 3LHPM-ICA and GC on the 67 training data. The extracted features were then entered into the classifier model. In the testing stage, the ICA was performed on the testing data. Then the most similar ICs of the testing data were selected by computing the Euclidean distance between the ICs of the testing data and the reproducible ICs from the training data. Finally, the ROIs of the selected ICs and GCIs were entered into the classifier for the prediction of AD/NC. For each subject, the 24 by 24 feature matrix was entered into the deep learning network. With LOOCV strategy, a classification accuracy of 95.59% with a sensitivity of 97.06% and a specificity of 94.12% was achieved. For comparison the classifiers, including LeNet5 (LeCun et al., 1998), the kernel support vector machine (SVM), the maximum uncertainty linear discriminant analysis (MDLA; Dai et al., 2012) and autoencoder (AE), were also performed. The deep neural network with stacked AEs consisted of five layers: an input layer, two hidden layers, a softmax layer and a classification layer. First, we trained the hidden layers individually in an unsupervised fashion using AEs. Then we trained a softmax layer and joined the layers together to form a stacked network. Finally, a supervised fine-tuning stage was applied to improve the classification performance by performing backpropagation on the whole multilayer network. The numbers of nodes were set to be 100 and 50 in the first and second hidden layers, respectively. A Gaussian kernel with a width of 0.5 was used in SVM. Several types of features, including the AAL atlas-based features, GC features and combined ICA and GC features with different classifiers were also implemented. The AAL atlas-based features were 90 × 90 matrices obtained by calculating the Pearson correlation coefficients between the brain regions, excluding the cerebellum, that were defined with AAL atlas. The upper triangular feature matrices were reshaped as feature vectors when SVM and MDLA were performed. The classification results are shown in Table 2. It can be seen that the classification performance of the DAG network combined with ICA and GC features is better than the values obtained with any single type of features or other types of classifiers.

**Table 2:** Classification performance of different methods with leave-one-out cross validation (LOOCV).

Methods	Accuracy	Sensitivity	Specificity
AAL atlas based+SVM	77.94%	73.53%	82.35%
AAL atlas based+MDLA	75.0%	79.41%	70.59%
AAL atlas based+LeNet5	79.41%	76.47%	82.35%
AAL atlas based+AE	80.88%	76.47%	85.29%
AAL atlas based+DAG	82.35%	79.41%	85.29%
GC+SVM	83.82%	85.29%	82.35%
GC+MDLA	82.35%	88.24%	76.47%
GC+LeNet5	85.29%	82.35%	88.24%
GC+AE	88.24%	82.35%	94.12%
GC+DAG	88.24%	91.18%	85.29%
ICA+GC+SVM	91.18%	88.24%	94.12%
ICA+GC+MDLA	89.71%	97.06%	82.35%
ICA+GC+LeNet5	92.65%	94.12%	91.18%
ICA+GC+AE	94.12%	97.06%	91.18%
ICA+GC+DAG	95.59%	97.06%	94.12%

**Figure 5:**
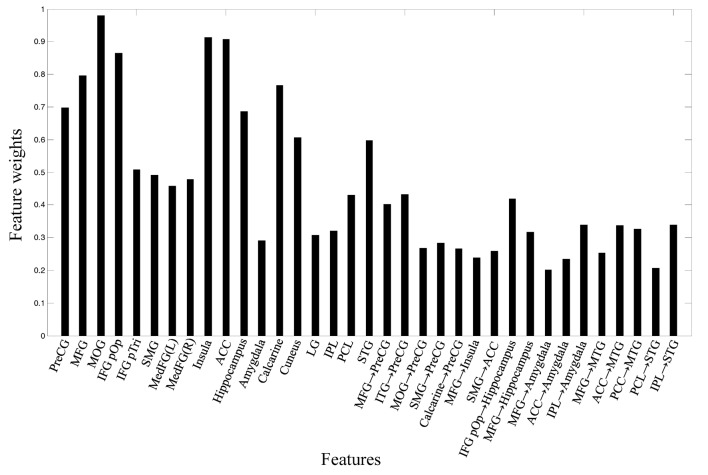
Feature weights in the classification.

The weights of the features were computed by the coefficients of the discrimination hyperplane, and the most discriminative features for classification are shown in Figure 5. The connections with the largest weights are the most informative. It can be seen that the IC activity in the MOG, IFG, MFG, ACC, insula, hippocampus, STG, and the effective connections from IFG to hippocampus, from ITG to precentral gyrus (PreCG), and from MFG to hippocampus made larger contributions to the classification.

## Discussion

In the current work, we presented a 3LHPM-ICA approach which addressed the problems in spatial individual ICA algorithm such as the uncertainty of the number of components, the randomness of initial values, and the correspondence of ICs among multiple subjects. Then, we applied the 3LHPM-ICA method and GC on resting state fMRI data to investigate the reproducible and stable ICs across individuals. We then obtained the intrinsic brain functional and effective connectivity feature matrices. A deep learning framework was finally investigated to assess if these brain features can serve as biomarkers for AD.

We found significantly decreased intrinsic FC in AD patients compared to NC in several subcortical regions including the hippocampus, amygdala, insula and putamen. As one of the earliest and most widely investigated brain regions in AD, researchers have correlated alterations in hippocampal activity and connectivity as well as shrinkage with the presence of AD, which explains one of the early symptoms in the impairment of memory, especially the formation of new memories in AD patients (Wang L. et al., 2006; Allen et al., 2007; Mu and Gage, 2011; Smith et al., 2014). Amygdala atrophy in AD and its relation to global illness severity have also been reported (Scott et al., 1991; Barnes et al., 2006; Poulin et al., 2011), elucidating the aberrant motor behavior, anxiety and irritability of AD patients. Another positron emission tomographic study of AD reported the cholinergic deficit in the amygdala, supporting that the amygdala played an important role in the retention of affective conditioning and/or memory consolidation and cross-verified the role of the amygdala in the emotional and behavioral symptoms of AD (Shinotoh et al., 2003). The insula is a key region for cognition, emotion and sensory processes which has been demonstrated with gray matter loss (Guo et al., 2012), abnormal activities (Lin et al., 2017), and disrupted connections in AD (Xie et al., 2012; Liu et al., 2018). Furthermore, the reduced volumes of putamen, which was correlated with impaired global cognitive performance, might contribute to cognitive decline in AD (de Jong et al., 2008; Roh et al., 2011). Consistent with the previous studies, our findings of decreased brain connectivity in certain subcortical areas indicated that these alterations might be related to the memory, emotion, motor and cognition disorders present in AD patients.

The loss of neurons and synapses in the cerebral cortex of AD results in gross atrophy of the affected regions, including degeneration in the temporal gyrus, parietal lobe, and parts of the frontal cortex and cingulate gyrus. Neuropathological studies have shown that AD-related degeneration begins in the medial temporal lobe (Braak and Braak, 1995). The current finding of decreased FC in the temporal gyrus is in line with previous reports of temporal gyrus atrophy (Farrow et al., 2007; Frisoni et al., 2010; Ho et al., 2010) and FC anomalies (Toussaint et al., 2014), leading to the memory and learning deficits that are classically observed with early clinical manifestations of AD. Our results also revealed disrupted resting state functional connectivities in the DMN, which consists of the PCC, inferior parietal lobe (IPL) and prefrontal cortex (PFC). The cortical thinning (Dickerson and Sperling, 2009) and decreased intrinsic brain activity (He et al., 2007; Wang et al., 2011b) and connectivity (Greicius et al., 2004; Toussaint et al., 2014) of DMN have been demonstrated in many studies. Therefore, our findings provide further evidence that the aberration of DMN may result in the episodic memory, visual imagery and mentalizing disorders in AD. Moreover, as part of the frontostriatal circuit which is composed of the ACC, PFC and parts of the basal ganglia, the ACC is involved in effort-based decision making and executive functions (Stella et al., 2014; Theleritis et al., 2014; Le Heron et al., 2018). Disruption of the FC in ACC found in this study might play a pivotal role in apathy, such as behavioral activation, social motivation and emotional sensitivity disorders in AD patients. Therefore, the brain connectivity alterations of the identified cortical and subcortical regions in this study may be associated with the cognitive and functional impairment of AD and potentially served as clinical biomarkers of AD.

The two-dimensional features fused by the FC obtained by 3LHPM-ICA and effective connectivity derived from GC were then applied for classification in this study. Compared with the traditional feature arrangement and fusion method, which usually reshaped the two dimensional features into a vector or concatenated different types of features into a longer feature vector (Wang K. et al., 2006; Chen et al., 2011; Dai et al., 2012; Dyrba et al., 2015; de Vos et al., 2018), the two dimensional feature matrices and feature fusion method used in this study preserved the spatial structural characteristics of features and provided a more meaningful way to combine various types of features for classification. Moreover, the overfitting issue, which may be caused by high-dimensional feature space in the traditional methods, could be alleviated due to the two dimensions of features in this study.

Advanced deep learning techniques have been successfully applied for the diagnosis of AD based on PET and sMRI (Suk and Shen, 2013; Liu S. et al., 2015; Ortiz et al., 2016; Lu et al., 2018; Shi et al., 2018). A recent report constructed a customized AE architecture with resting-state correlation based FC to classify mild cognitive impairments from NCs (Ju et al., 2017). However, different parcellation schemes may generate different results. Therefore, compared with the correlation-based method, the data-driven method in this study avoided the problem whereby the brain parcellation methods may affect classification performance. The connectivity patterns of brain networks derived from ICA and GC were stable and not influenced by different parcellation atlases. Moreover, we compared two kinds of deep learning algorithms with the same inputted features. One was LeNet5 with sequential connected layers and the other was the DAG network, which consisted of sequential connected layers and shortcut connections. Our results demonstrated that the DAG network has better performance than the sequential network, possibly because of the “skip” connections between layers with feed-forward computations.

Several limitations of the present study should be noted. First, the sample size in this study was not large and future work should be done on a larger training sample in order to improve the robustness and generalization of the classification model. Second, multimodal neuroimaging features such as sMRI and DTI should also be investigated in addition the resting state fMRI, which may lead to higher classification accuracy. Third, we used a binary classification for the prediction of AD/NC. However, multi-class classification should be considered for its clinical applications in the future because there are different stages of AD such as MCI, LMCI and EMCI. Fourth, it would be more comparable to compare the accuracy results with the same benchmark datasets. Therefore, future work will focus on the implementation of different models based on public datasets such as ADNI. Finally, a light deep architecture with two-dimensional input images was applied in this study. More complicated deep learning models should be implemented such as GoogLeNet, AlexNet, VGG, ResNet and 3D convolutional neural networks, which may be more appropriate for big data. Nevertheless, our results suggested that the functional and effective connectivity features extracted by 3LHPM-ICA and GC followed by deep learning classification represented the most powerful method of distinguishing AD from healthy data. Due to the flexibility of this technique, it has the potential to be extended to other psychiatric disorders in the future.

## Author Contributions

JQ, YL, and AL conceived and designed the experiments and performed the experiments. JQ, ZW and AL analyzed the data and contributed reagents, materials and analysis tools. JQ, CC, and AL wrote the article.

## Conflict of Interest Statement

The authors declare that the research was conducted in the absence of any commercial or financial relationships that could be construed as a potential conflict of interest.
